# Temperate performance and metabolic adaptations following endurance training performed under environmental heat stress

**DOI:** 10.14814/phy2.14849

**Published:** 2021-05-12

**Authors:** Ed Maunder, Daniel J. Plews, Gareth A. Wallis, Matthew J. Brick, Warren B. Leigh, Wee‐Leong Chang, Casey M. Watkins, Andrew E. Kilding

**Affiliations:** ^1^ Sports Performance Research Institute New Zealand Auckland University of Technology Auckland New Zealand; ^2^ School of Sport, Exercise, and Rehabilitation Sciences University of Birmingham Birmingham UK; ^3^ Orthosports North Harbour AUT Millennium Auckland New Zealand; ^4^ Faculty of Health and Environmental Sciences Auckland University of Technology Auckland New Zealand

**Keywords:** adaptation, endurance training, heat stress, mitochondria, performance

## Abstract

Endurance athletes are frequently exposed to environmental heat stress during training. We investigated whether exposure to 33°C during training would improve endurance performance in temperate conditions and stimulate mitochondrial adaptations. Seventeen endurance‐trained males were randomly assigned to perform a 3‐week training intervention in 18°C (TEMP) or 33°C (HEAT). An incremental test and 30‐min time‐trial preceded by 2‐h low‐intensity cycling were performed in 18°C pre‐ and post‐intervention, along with a resting *vastus lateralis* microbiopsy. Training was matched for relative cardiovascular demand using heart rates measured at the first and second ventilatory thresholds, along with a weekly “best‐effort” interval session. Perceived training load was similar between‐groups, despite lower power outputs during training in HEAT versus TEMP (*p* < .05). Time‐trial performance improved to a greater extent in HEAT than TEMP (30 ± 13 vs. 16 ± 5 W, *N* = 7 vs. *N* = 6, *p* = .04), and citrate synthase activity increased in HEAT (fold‐change, 1.25 ± 0.25, *p* = .03, *N* = 9) but not TEMP (1.10 ± 0.22, *p* = .22, *N* = 7). Training‐induced changes in time‐trial performance and citrate synthase activity were related (*r* = .51, *p* = .04). A group × time interaction for peak fat oxidation was observed (Δ 0.05 ± 0.14 vs. −0.09 ± 0.12 g·min^−1^ in TEMP and HEAT, *N* = 9 vs. *N* = 8, *p* = .05). Our data suggest exposure to moderate environmental heat stress during endurance training may be useful for inducing adaptations relevant to performance in temperate conditions.

## Graphical abstract

We observed that 3 weeks of endurance training performed in 33°C improved endurance performance in temperate conditions to a significantly greater extent than matched training performed in 18°C. We also observed a significant increase in *vastus lateralis* citrate synthase activity in response to training performed in 33°C, but not matched training performed in 18°C.

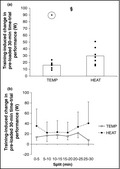

**New findings**

*What is the central question of this study?*

We investigated the hypothesis that a 3‐week endurance training intervention performed under moderate environmental heat stress would improve endurance performance in temperate conditions and metabolic adaptations to training compared to matched training performed in temperate conditions.

*What is the main finding and its importance?*

We observed that 3 weeks of endurance training performed in 33°C improved endurance performance in temperate conditions to a significantly greater extent than matched training performed in 18°C. We also observed increased *vastus lateralis* citrate synthase activity after training performed in 33°C, but not matched training performed in 18°C. These data suggest exposure to moderate environmental heat stress during a 3‐week block of training may be favorable for endurance cyclists.

## 1 INTRODUCTION

A goal of endurance training is to induce physiological adaptations that improve determinants of endurance performance such as maximum oxygen uptake (${\dot{V}O}_{2}\text{max}$), physiological thresholds, and exercise economy (Holloszy, 2008). Endurance athletes competing in stochastic, drafting events such as road cycling (Lucía et al., 1999) would likely also benefit from adequate metabolic flexibility to effectively utilize fat as an energy substrate at given submaximal workloads in order to preserve finite endogenous carbohydrate stores, alongside well‐developed, rapid carbohydrate metabolism to facilitate short periods of intense effort (Hawley et al., 2018). These adaptations are at least partly mediated through effects on skeletal muscle mitochondrial content and function, which have been shown to adapt in response to endurance training (Granata et al., 2016a, 2016b; Hoppeler et al., 1985; Montero et al., 2015; Scalzo et al., 2014; Spina et al., 1996). Thus, a key focus of endurance training is to improve mitochondrial content and function in order to favorably impact endurance performance.

Many endurance athletes undertake training camps in hot environments (Maunder, Kilding, et al., 2020) in an effort to increase the stimulus for training adaptation (Hawley et al., 2018). Specifically, endurance training performed under heat stress may upregulate expression of various factors implicated in adaptive signaling cascades coordinating mitochondrial biogenesis. These include expression of heat shock proteins (Skidmore et al., 1995) and interleukin‐6 (Starkie et al., 2005) in muscle, circulating catecholamines (Febbraio et al., 1994; Hargreaves et al., 1996), muscle glycogenolysis (Febbraio et al., 1994), and muscle and plasma lactate accumulation (Febbraio et al., 1994; Maunder, Plews, et al., 2020). However, the consequences of endurance training performed under environmental heat stress for adaptations to skeletal muscle mitochondria and metabolism have not been investigated using longitudinal designs. In vivo investigation is limited to one acute study, which observed blunted transcription of genes related to mitochondrial biogenesis immediately and 3 h post‐matched work‐rate‐exercise performed in 33°C compared to 20°C (Heesch et al., 2016). These results were in contrast to the authors' hypothesis, which was based on in vitro studies reporting positive effects of heat exposure for acute and chronic mitochondrial changes (Liu & Brooks, 2012; Patton et al., 2018; Tamura & Hatta, 2017), and the basic training principle that greater homeostatic disturbances lead to greater training adaptations (Fiorenza et al., 2018; Hawley et al., 2018). Accordingly, there has been no investigation of mitochondrial adaptations to a heat stress training intervention in endurance athletes.

Studies investigating the effect of endurance training performed under heat stress for functional adaptations relevant to performance in temperate conditions have produced mixed results (Karlsen et al., 2015; Keiser et al., 2015; Lorenzo et al., 2010; Mikkelsen et al., 2019; Waldron et al., 2019). Several studies have reported favorable hematological adaptations with exercise training performed in hot environments (McCleave et al., 2017, 2020; Rønnestad et al., 2020). When training under heat stress, a reduced absolute power output is achieved for a given heart rate (HR) (Nybo & Nielsen, 2001; Rowell et al., 1966) and physiological threshold (James et al., 2017; Lorenzo et al., 2010), likely resulting in reduced mechanical work‐rates during training (Boynton et al., 2019; Drust et al., 2005). The addition of heat stress may therefore induce a reduced mechanical load compared to training performed in temperate conditions with equivalent cardiovascular demand or perceived exertion. However, previous studies in this domain have not accounted for this, with training programs matched for absolute work‐rates (McCleave et al., 2017, 2020; Rønnestad et al., 2020), performed in an uncontrolled setting (Karlsen et al., 2015), and/or using training programs not reflective of real‐world endurance sport (Keiser et al., 2015; Lorenzo et al., 2010; Mikkelsen et al., 2019; Waldron et al., 2019). Accordingly, there has so far been little systematic evaluation of the adaptive metabolic or temperate performance effects of exposure to environmental heat stress during endurance training in well‐matched training programs.

Therefore, the aim of the present investigation was to determine the effect of a three‐week endurance training intervention performed in 33°C on metabolic adaptations and performance in temperate conditions, in comparison to an appropriately matched training program performed in 18°C. It was hypothesized that exposure to 33°C during training would augment training‐induced changes in *vastus lateralis* citrate synthase activity and fat oxidation during exercise and therefore improve pre‐loaded time‐trial (TT) performance in temperate conditions to a greater extent than matched training performed in 18°C.

## 2 METHODS

### 2.1 Ethical approval

This study was performed in accordance with the standards of the Declaration of Helsinki, 2013. The Auckland University of Technology Ethics Committee approved all procedures (19/146), and all participants provided written informed consent prior to participation.

### 2.2 Participants

A priori sample size estimation indicated nine participants per group were required to detect a statistically significant training‐induced increase in whole‐muscle fat transporter protein content using data from a three‐week exercise training intervention in a similar endurance‐trained cohort with 80% statistical power (Hulston et al., 2010). Seventeen endurance‐trained male cyclists and triathletes took part in the present investigation (age, 34 ± 7 y; height, 181 ± 8 cm; mass, 80.5 ± 9.6 kg; sum of eight skinfolds, 71 ± 29 mm; recent training volume, 8 ± 2 h·week^−1^; maximum oxygen uptake [${\dot{V}O}_{2}\text{max}$], 4.3 ± 0.7 L·min^−1^). Three participants were in‐progress when the first nationwide lock‐down for COVID‐19 in New Zealand was announced; hence, some post‐testing data in three participants (*N* = 2 in the heat group, *N* = 1 in the temperate group) were not successfully collected. The subsequent regional lock‐down prevented collection of a larger sample size. The actual sample size for outcome measures is indicated in the relevant section. This study was conducted during a maintenance phase of training in all participants.

### 2.3 Study design

**FIGURE 1:**
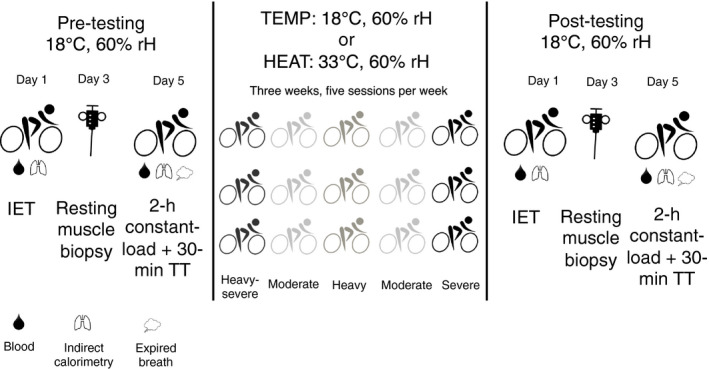
Schematic representation of the study design

The present investigation adopted a randomized controlled trial design, and was conducted in the non‐summer months in Auckland, New Zealand (Figure 1). The initial laboratory visit involved an incremental, maximal exercise test performed on a cycle ergometer in temperate conditions (18°C, 60% relative humidity [rH]). This assessment was used to ascertain a physiological profile, including HR at individual physiological thresholds in order to individualize subsequent training interventions. Following the incremental test, participants completed a 30‐min familiarization time‐trial ahead of subsequent experimental TTs. An overnight‐fasted, resting muscle microbiopsy was then performed during a separate visit ~48 h following the incremental test. Participants returned to the laboratory, ~48 h later, to complete the pre‐intervention experimental TT on a cycle ergometer in temperate conditions (18°C, 60% rH). Participants subsequently commenced a 3‐week training program, having been randomly allocated to either a temperate (TEMP, 18°C, 60% rH) or moderate environmental heat stress (HEAT, 33°C, 60% rH) intervention group prior to the initial assessment using a random number generator (random.org). During the training intervention serial measures of waking HRV and subjective well‐being were made. Following the training program, the incremental exercise test, resting muscle microbiopsy, and experimental TT were repeated.

### 2.4 Incremental exercise tests

Participants arrived for the incremental exercise test at ~07:00 having fasted overnight and refrained from alcohol and vigorous exercise for 24 h. Height and body mass were then recorded prior to the incremental cycling test. Cycling began at 95 W and the work rate increased by 35 W every 3 min (Excalibur Sport, Lode), with continuous collection of expired gases using indirect calorimetry (TrueOne2400, ParvoMedics) and HR (RS800, Polar Electro Oy). Fingertip capillary blood samples were obtained at the end of each 3‐min stage and analyzed for lactate concentration using an automatic analyzer (Lactate Pro 2, Arkray). When blood lactate concentration exceeded 4.0 mmol·L^−1^, step duration was reduced to 1 min until attainment of volitional exhaustion. ${\dot{V}O}_{2}\text{max}$ was accepted as the highest 15‐s average oxygen consumption providing two of the following three criteria were met: respiratory exchange ratio >1.10, HR ±10 b·min^−1^ of the age‐predicted maximum HR (205.8 − 0.685[age (y)]) (Inbar et al., 1994), and attainment of volitional exhaustion. The first ventilatory threshold (VT_1_) was identified as the work rate at which the ventilatory equivalent for oxygen ($\dot{V}E\cdot {\dot{V}O}_{2}^{‐1}$) began to increase in the absence of changes in the ventilatory equivalent for carbon dioxide ($\dot{V}E\cdot {\dot{V}\text{CO}}_{2}^{‐1}$), the second ventilatory threshold (VT_2_) was identified as the first work rate at which $\dot{V}E\cdot {\dot{V}O}_{2}^{‐1}$ and $\dot{V}E\cdot {\dot{V}\text{CO}}_{2}^{‐1}$ increased alongside a reduction in PetCO_2_ (Lucía et al., 2000). Two blinded expert observers determined ventilatory thresholds. Power output at 2 and 4 mmol·L^−1^ blood lactate concentrations were calculated using available software (Lactate Dashboard 1.1.1, https://shiny.fmattioni.me/lactate/), which hosts R code for the calculation of lactate threshold parameters described in recent work (Jamnick et al., 2018). Following the incremental exercise tests, participants rested for ~20 min before completing a self‐paced 30‐min TT, to act as familiarization prior to the experimental TT. Following completion of the familiarization TT, participants were instructed on how to use a smartphone application for measurement of waking HRV (HRV4Training), and instructed to complete it each morning for the duration of the study. Participants were provided with “base” carbohydrate (e.g., pasta, rice, noodles, rolled oats) to provide 1 g·kg^−1^ for dinner at ~20:00 the evening before, and 1 g·kg^−1^ for breakfast two hours prior to, the experimental TT. Participants were instructed to record any additions they made to the base carbohydrate for these meals, photograph their prepared and finished meal, and send these photographs to the primary researcher such that these meals could be replicated precisely in advance of the post‐training experimental TT. Lastly, participants were instructed to precisely record all exercise they performed throughout the pre‐training assessment week, such that this could be repeated in the post‐training assessment week. The replication of these training records was verified by the primary researcher.

### 2.5 Resting muscle microbiopsy

Participants arrived at the laboratory ~48 h following the incremental exercise test at ~09:00 having fasted overnight. A muscle microbiopsy was then obtained from the *vastus lateralis* using the microbiopsy technique (Hayot et al., 2005). Briefly, local anesthesia was applied to the skin and superficial muscle fascia, after which a microbiopsy needle was inserted into the mid‐belly of the *vastus lateralis* to a depth of ~2 cm to recover ~15 to 20 mg of tissue using a spring‐loaded mechanism (14G Ultimate Biopsy Needle, Zamar Care). Muscle tissue was immediately frozen using dry ice, and stored at −80°C until further analysis. The site of the pre‐training microbiopsy was recorded by an International Society of Kinathropometry accredited anthropometrist such that the post‐training microbiopsy occurred ~2 cm from the site of the pre‐training biopsy.

### 2.6 Time‐trial performance assessments

Participants arrived at the laboratory for performance TTs at ~08:00, having been asked to avoid high‐^13^C foodstuffs (e.g., corn‐derived sources) and vigorous exercise for 48 h, and having ingested their standardized pre‐trial dinner at ~20:00 the previous evening and standardized pre‐trial breakfast ~2 h beforehand. Sum of eight skinfold thickness was then determined by an International Society of Kinathropometry accredited anthropometrist (triceps brachii, biceps brachii, subscapular, iliac crest, supraspinale, abdominal, anterior thigh, posterior shank). Participants were fitted with an antecubital venous cannula, from which a 5‐ml resting blood sample was drawn, and HR monitor (RS800, Polar Electro Oy). Resting expired breath samples were collected into duplicate 10‐ml evacuated, plain tubes to correct exercise samples for background expired ^13^C.

Cycling then commenced for 2 h in a temperature‐controlled laboratory (18°C and 60% rH) on an ergometer (Excalibur Sport, Lode) at 80% of the work rate eliciting VT_1_ in the pre‐training IET. Convective airflow was provided by an industrial fan (FS‐75, FWL). Participants consumed 60 g·h^−1^ of glucose in 7.5% liquid solutions at 15‐min intervals throughout the constant‐load phase. Beverages were enriched with ~47 mg·L^−1^ of isotopically labeled [U‐^13^C]glucose (Cambridge Isotope Laboratories), such that the ingested solution was high in ^13^C (~50 δ‰ vs. Pee Dee Bellemnitella [PDB]). Expired gases were collected for 4 min every 15 min using a metabolic cart (TrueOne2400, ParvoMedics), 5‐ml venous blood samples were obtained every 30 min, HR was recorded continuously, and expired breath samples were collected into duplicate 10‐ml evacuated plain tubes at 60, 90, and 120 min.

Upon completion of the 2‐h constant‐load phase, participants commenced a 30‐min, maximal‐effort TT (IndoorTrainer, SRM). During the TT, participants were blinded to power and HR, but informed of the time remaining every 10 min and with 5 and 1‐min remaining. Participants were instructed to produce as much power as possible over the 30 min, and average power output was used as the performance measure.

### 2.7 Training interventions

Following completion of the pre‐intervention TT, participants began an individualized 3‐week training program, having being randomly allocated to either a temperate (TEMP) or moderate environmental heat stress (HEAT) training intervention. Training interventions were designed with input from an expert practitioner working with elite endurance athletes, with the stated goal of the training program design to improve pre‐loaded 30‐min TT performance. Training interventions were based on HR at the ventilatory thresholds in the pre‐training IET (Table 1), and included five supervised training sessions in the laboratory each week in either 18°C (TEMP) or 33°C (HEAT), both at 60% rH, for a total of 15 supervised training sessions during the three‐week intervention. Sessions were performed in the order shown in Table 1. Water was consumed ad libitum and convective fan cooling was provided during all training sessions. Training sessions were programmed according to HR measured at ventilatory thresholds obtained in the pre‐training IET such that the relative cardiovascular demand of the training program was equal between‐subjects and between‐groups. When attempting to match for relative cardiovascular demand, data collected in our laboratory indicate that measures of HR at individual physiological thresholds are stable between temperate and moderate heat stress environments, despite reductions in absolute power outputs at those thresholds (Maunder et al., 2021). Therefore, there was no requirement for an IET to be performed under heat stress to derive these measurements for HEAT.

**TABLE 1:** Training intervention in the present investigation, which took place in 18°C (TEMP) or 33°C (HEAT) and 60% relative humidity

	Type	Session
1	Heavy‐severe	4–6 × 8 min at HR at VT_2_, 2 min recovery
2	Moderate	90 min at 95% of HR at VT_1_
3	Heavy	3 × 25 min at midpoint of HR at VT_1_ and VT_2_, 5 min recovery
4	Moderate	90 min at 95% of HR at VT_1_
5	Severe	6–10 × 3 min at “best session effort,” 2‐min recovery

These five sessions were all performed each week, in the order shown. Where a range of repetitions is shown (heavy‐severe and severe‐intensity sessions), a uniform progression in the number of repetitions over the three weeks was used. Heart rate at the first (VT_1_) and second (VT_2_) ventilatory thresholds was quantified in the pre‐intervention incremental exercise test.

Abbreviations: HR, heart rate, VT_1_, first ventilatory threshold, VT_2_, second ventilatory threshold.

Subjective wellness using 5‐point Likert scale questionnaires assessing fatigue, sleep quality, muscle soreness, stress, and mood (McLean et al., 2010) were assessed prior to each training session, along with the duration and perceived exertion of any additional training performed since the previous session using Borg's 1–10 scale (Borg, 1998). Participants were asked to limit training outside of the study to a maximum of 3 h per week, all at low intensity. This was considered necessary to allow training adaptations from swimming, running, and resistance exercise to be maintained, thus reducing the possibility of detraining in other exercise modalities impacting cycling performance. Following each training session, duration, power, HR, and perceived session exertion using Borg's 1–10 scale were recorded. Training load was calculated using the session‐RPE model (Christen et al., 2016). Daily HRV measurements, expressed as the root mean square of the sum of successive differences in R‐R intervals (rMSSD), were extracted from the previously validated (Plews et al., 2017) smartphone application in which they were collected (HRV4Training). Mean and standard deviation (SD) absolute HRV values for each training week were calculated for each participant. Day‐to‐day variation in HRV measurements was calculated for each participant in each training week by expressing the weekly SD as a percentage of the weekly mean (Flatt & Howells, 2019; Javaloyes et al., 2019).

### 2.8 Muscle analysis

Frozen muscle samples were rinsed using cold phosphate‐buffered saline (PBS) and ~5 mg samples were suspended to 25 mg·ml^−1^ in PBS and ground manually using a precooled glass Dounce homogenizer. Resulting homogenate was solubilized with extraction buffer (ab260490, Abcam®) to 5 mg·ml^−1^ and incubated on ice for 20 min prior to centrifugation at 16,000 *g* for 20 min at 4°C. Supernatant was extracted and stored at −80°C prior to further analysis. A Bradford assay for sample protein concentration was subsequently performed in duplicate (coefficient of variation [CV], 3.7%). Briefly, a Coomassie blue G reagent was added to protein standards and samples, and optical density was measured on a spectrophotometer at 595 nm (ab102535, Abcam®). Maximal citrate synthase (CS) activity was measured via a kinetic immunocapture assay (ab119692, Abcam®). The activity of CS captured within microplate wells was determined via colour development of TNB, generated from DTNB present in the citrate synthase reaction, via measurement of optical density at 412 nm every 30 s for 10 min. The slope of the optical density versus time curve was blank‐corrected and used to estimate CS activity. Cluster of differentiation 36 (CD36) concentration was measured via an enzyme‐linked immunosorbent assay (ab267614, Abcam®). CD36 was captured within microplate wells by an immobilized antibody. After washing, a biotinylated anti‐human CD36 antibody was added to individual wells, washed, and HRP‐conjugated streptavidin was added. Wells were washed again prior to addition of a TMB substrate solution. Color development proportional to CD36 concentration was measured after addition of a stop solution at 450 nm. These assays were performed using commercially available kits (as indicated) in duplicate on a spectrophotometer (Multiskan GO, Thermo Fisher Scientific Inc.) according to the manufacturer's instructions. Achieved CVs were 5.6% for CS activity and 9.8% for CD36 concentration, both expressed relative to total muscle protein concentration.

### 2.9 Gas analysis

Indirect calorimetry was performed using a metabolic cart (TrueOne2400, ParvoMedics). Oxygen uptake (${\dot{V}O}_{2}$) and carbon dioxide production (${\dot{V}\text{CO}}_{2}$) values were averaged for the final 1‐min of each step during the incremental exercise tests, and 3‐min prior to each measurement time‐point during the TT. Whole‐body rates of energy expenditure, carbohydrate oxidation, and fat oxidation were calculated according to standard non‐protein equations (Equation 1) (Jeukendrup & Wallis, 2005). Exogenous (CHO_exo_) and endogenous (CHO_end_) carbohydrate oxidation rates during the 60–120 min constant‐load phase of the experimental trials were estimated using gas chromatography continuous‐flow mass spectrometry (Equation 2) and standard equations (Equation 3) (Craig, 1957). Gross cycling efficiency was calculated as the percentage of energy expenditure converted into mechanical work (power) (Moseley & Jeukendrup, 2001).

(1) $$\begin{array}{cc} \text{Energy expenditure}\left(\text{kcal}\cdot \min^{‐1}\right) & =\left(0.55\times {\dot{V}\text{CO}}_{2}\right)+\left(4.471\times {\dot{V}O}_{2}\right)\\ \text{Carbohydrate oxidation}\left(g\cdot \min^{‐1}\right) & =\left(4.21\times {\dot{V}\text{CO}}_{2}\right)‐\left(2.962\times {\dot{V}O}_{2}\right)\\ \text{Fat oxidation}\left(g\cdot \min^{‐1}\right) & =\left(1.695\times {\dot{V}O}_{2}\right)‐\left(1.701\times {\dot{V}\text{CO}}_{2}\right) \end{array}$$

where ${\dot{V}O}_{2}$ and ${\dot{V}\text{CO}}_{2}$ are in L·min^−1^.

(2) $$\delta^{13}C=\left[\left({\;}^{13}C:{\;}^{12}\text{C sample}/{\;}^{13}C:{\;}^{12}\text{C standard}\right)‐1\right]\times 10^{3}{\text{mL}}^{‐1}$$

where isotopic enrichment was expressed as δ·ml^−1^ and related to an international standard (PDB).

(3) $$\begin{array}{cc} {\text{CHO}}_{\text{exo}} & ={\dot{V}\text{CO}}_{2}\times \left[\left(\delta \exp‐\exp_{\text{bkg}}\right)/\left(\delta \text{Ing}‐\exp_{\text{bkg}}\right)\right]\;\times \left(1/0.7467\right)\\ {\text{CHO}}_{\text{end}} & ={\text{CHO}}_{\text{tot}}‐{\text{CHO}}_{\text{exo}} \end{array}$$

where δExp = ^13^C enrichment of expired gas sample, δIng = ^13^C enrichment of ingested carbohydrate, Exp_bkg_ = ^13^C enrichment of expired gas sampled at rest, $0.7467={\dot{V}\text{CO}}_{2}$ of 1 g glucose oxidation, and CHO_tot_ = total carbohydrate oxidation rate.

### 2.10 Statistical analysis

Sample data are expressed mean ± SD unless otherwise stated. Primary outcome measures were TT performance and muscle adaptations, and whole‐body fat and CHO oxidation rates during exercise. Two‐way analyses of variance were used to assess within‐group, between‐group, and interaction effects in measured variables, with the location of significant effects detected post‐hoc using Holm–Bonferroni stepwise correction. Specific pre‐planned within‐ and between‐group comparisons were also made using paired *t*‐tests for primary outcome measures, along with Pearson's correlation coefficients and Cohen's *d* effect sizes (ES) where appropriate. Significance was inferred when *p* ≤ .05.

## 3 RESULTS

### 3.1 Training interventions

**FIGURE 2:**
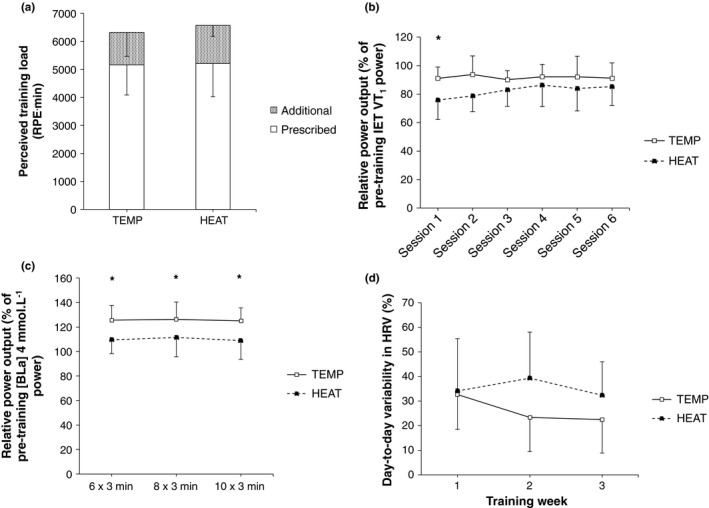
Characteristics of the 3‐week training interventions in TEMP (*N* = 8) and HEAT (*N* = 9). (a) RPE‐time training load, (b) power output during the 90‐min constant heart rate moderate‐intensity training sessions relative to power output at the first ventilatory threshold (VT_1_) in the pre‐training incremental exercise test, (c) average 3‐min repetition power output during the 6 × 3 min, 8 × 3 min, and 10 × 3 min severe‐intensity training sessions relative to power output at 4 mmol·L^−1^ blood lactate concentration in the pre‐training incremental exercise test, and (d) coefficient of variation in weekly resting heart rate variability (rMSSD) in the three training weeks. ‘*’ indicates *p* ≤ .05 between‐groups

All the subjects completed all the 15 prescribed training sessions. Total (*p* = .67), prescribed (*p* = .92), and additional (*p* = .54) RPE‐based training load did not significantly differ between‐groups (Figure 2a). Corrected pairwise comparisons revealed power output achieved during the moderate‐intensity training sessions, expressed relative to each individual's power at VT_1_ in the pre‐training IET, was significantly lower in HEAT versus TEMP in session one (76 ± 14 vs. 91 ± 8%, *p* = .04), and approached significance in session two (79 ± 11 vs. 94 ± 13%, *p* = .06, Figure 2b). Average power output during the 3‐min “best effort” repetitions in the severe‐intensity training sessions, expressed relative to each individual's power at 4 mmol·L^−1^ blood lactate concentration in the pre‐training IET, was significantly lower in HEAT versus TEMP (*p* = .03, Figure 2c). No significant between‐ or within‐group differences were observed for overall or weekly absolute HRV. Differences in day‐to‐day variation in HRV were not significant between‐groups (29 ± 17 vs. 39 ± 15% in TEMP and HEAT, respectively, *p* = .20) although this comparison approached significance in the second training week (23 ± 14 vs. 39 ± 19% in TEMP and HEAT, respectively, *p* = .08, Figure 2d). Self‐report well‐being data throughout the training period did not differ between‐groups (17.3 ± 1.5 vs. 16.9 ± 1.1 AU in TEMP and HEAT, respectively, *p* = .55).

### 3.2 Performance metrics

A Grubb's test revealed a significant outlier for training‐induced change in pre‐loaded 30‐min TT performance in TEMP (+90 W, Z = 2.23, *p* < .05). This participant achieved less power in their pre‐training TT than in all of their 90‐min moderate‐intensity training sessions (~−22 W), and with a ~14 b·min^−1^ lower HR than in their post‐training TT. They also vocally expressed following the pre‐training TT that they did not exert a maximal‐effort. Accordingly, this participant's TT performance data have been excluded. Inclusion of this erroneous data‐point would have abolished differences in training‐induced changes in time‐trial performance between‐groups.

**FIGURE 3:**
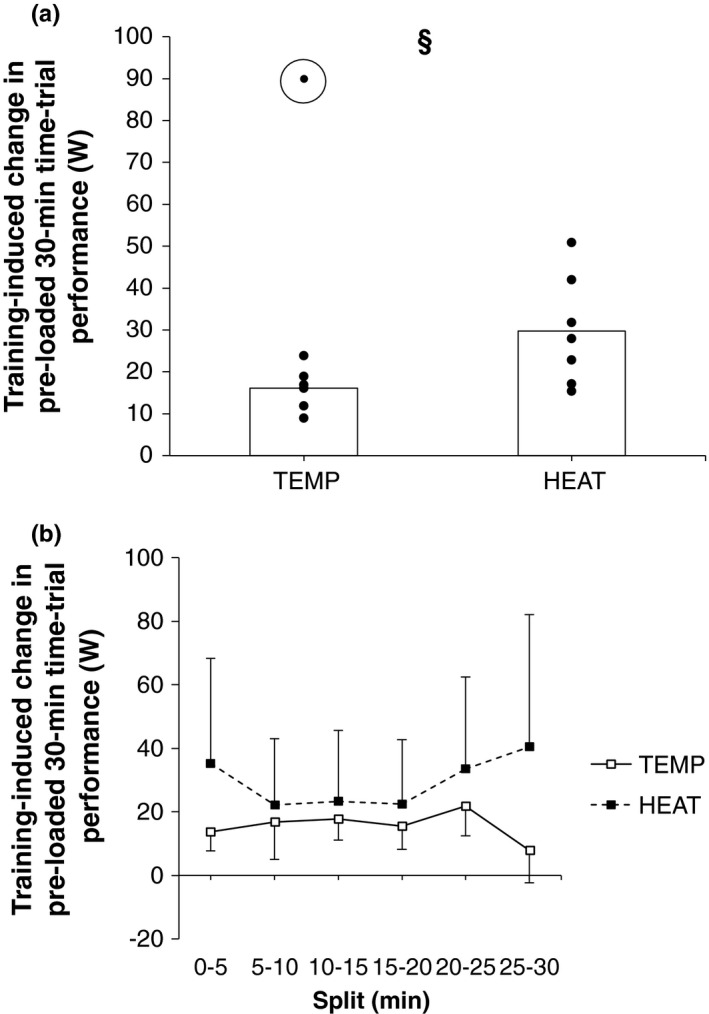
Training‐induced changes (a) average overall and (b) split power output during the pre‐loaded 30‐min time‐trial performance assessment in TEMP (*N* = 6) and HEAT (*N* = 7). Bars indicate group mean changes and dots indicate the results of individual participants. The erroneous data‐point not included in the analysis is circled in Figure 3a. ‘§’ indicates *p* ≤ .05 between‐groups

Pre‐loaded 30‐min TT significantly improved pre‐to‐post training in both groups, but to a significantly greater magnitude in HEAT (30 ± 13 vs. 16 ± 5 W, *p* = .04, Figure 3a, Table 2). Differences in magnitudes of training‐induced changes in average power during individual 5‐min splits were not significant between‐groups (*p* > .05, Figure 3b). Significant main effects for time were observed for power at 2 and 4 mmol·L^−1^ blood lactate concentration, VT_1_, and VT_2_, but significant group × time interactions were not observed (Table 2). Individual between‐group pairwise comparisons for training‐induced changes in these IET variables were also not significant (*p* > .07).

**TABLE 2:** Performance indicators with statistical comparisons

	Temperate training group				Heat training group				ANOVA		
	Pre	Post	Δ	ES	Pre	Post	Δ	ES	Group	Time	G × T
30‐min TT (W)	253 ± 54	269 ± 57^a^	16 ± 5^b^	0.25 ± 0.14	240 ± 38	270 ± 47^a^	30 ± 13^b^	0.69 ± 0.37	0.813	<0.001	0.038
B[La^−^] 2 mmol·L^−1^ (W)	192 ± 62	203 ± 61^a^	12 ± 14	0.10 ± 0.29	198 ± 66	226 ± 50^a^	28 ± 33	0.61 ± 0.65	0.618	0.008	0.221
B[La^−^] 4 mmol·L^−1^ (W)	241 ± 53	251 ± 54^a^	9 ± 8	0.18 ± 0.23	256 ± 55	275 ± 58^a^	19 ± 20	0.49 ± 0.40	0.472	0.002	0.219
VT_1_ (W)	190 ± 48	197 ± 34	7 ± 11	0.07 ± 0.39	191 ± 48	213 ± 44^a^	22 ± 21	0.45 ± 0.53	0.657	0.003	0.082
VT_2_ (W)	232 ± 45	251 ± 48^a^	20 ± 8	0.46 ± 0.29	253 ± 56	276 ± 59^a^	23 ± 18	0.48 ± 0.45	0.388	<0.001	0.590
${\dot{V}O}_{2}\text{max}$ (L·min^−1^)	4.25 ± 0.81	4.29 ± 0.82	0.04 ± 0.15	0.03 ± 0.24	4.29 ± 0.70	4.31 ± 0.66	0.02 ± 0.16	−0.03 ± 0.22	0.930	0.465	0.800

Significance is inferred when *p* ≤ .05. Cohen's *d* effect sizes (ES) are presented ±95% confidence limits. Data derived from the incremental exercise tests feature nine subjects in the temperate training group and eight subjects from the heat training group. Performance time‐trial data compares six subjects from the temperate training group and seven subjects from the heat training group.

Abbreviations: ${\dot{V}O}_{2}\text{max}$, maximum oxygen consumption; 30‐min TT, pre‐loaded 30‐min time‐trial; B[La^−^], blood lactate concentration; VT_1_, first ventilatory threshold; VT_2_, second ventilatory threshold.

^a^ Indicates significantly different versus pre‐training.

^b^ Indicates significantly different between‐groups.

### 3.3 Metabolic responses

**FIGURE 4:**
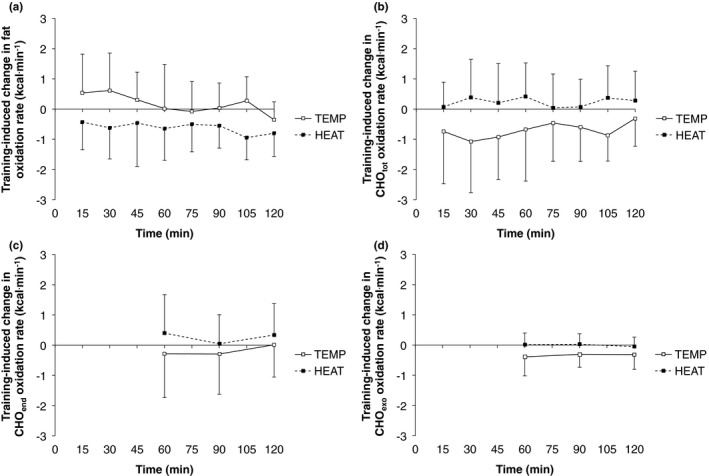
Training‐induced changes in (a) fat, (b) total carbohydrate (CHO_tot_), (c) endogenous carbohydrate (CHO_end_), and (d) exogenous carbohydrate (CHO_exo_) oxidation rates during the 120‐min constant‐load phase of the time‐trial. For all variables 7 subjects are included in the temperate and heat training groups

During IET, a significant group × time interaction was observed for the peak fat oxidation rate (PFO), whereby the training‐induced change in PFO was significantly lower in HEAT versus TEMP (Table 3). During the constant‐load phase of the TT, gross efficiency (GE) significantly improved in TEMP, but not HEAT, although the group × time interaction was not significant (Table 3). Significant group × time interactions were not observed for fat, total CHO, endogenous CHO, or exogenous CHO oxidation rates (Figure 4). Significant main effects of time were observed for mean HR during, and HR at 120 min of, the constant‐load phase of the TT, though no significant group × time interactions were observed (Table 3). Mean HR during the 30‐min TT was not significantly different between pre‐ and post‐training tests in either group (*p* > .10).

**TABLE 3:** Metabolic data with statistical comparisons

	Temperate training group				Heat training group				ANOVA		
	Pre	Post	Δ	ES	Pre	Post	Δ	ES	Group	Time	G × T
GE (%)	19.7 ± 1.1	20.6 ± 1.2^a^	1.0 ± 0.9	0.88 ± 0.71	18.9 ± 0.9	19.6 ± 0.7	0.7 ± 0.9	0.84 ± 1.17	0.080	0.006	0.574
Fat oxidation (g)	36 ± 23	39 ± 26	3 ± 10	0.21 ± 0.71	40 ± 19	33 ± 23	−7 ± 11	−0.26 ± 0.73	0.936	0.447	0.104
CHO_tot_ (g)	254 ± 38	233 ± 39	−22 ± 36	−0.69 ± 1.69	228 ± 68	235 ± 50	6 ± 29	0.01 ± 0.53	0.650	0.411	0.139
Mean CHO_exo_ (g·min^−1^)	0.63 ± 0.08	0.55 ± 0.13	−0.08 ± 0.12	−1.49 ± 2.13	0.49 ± 0.06	0.49 ± 0.06	0.00 ± 0.07	0.34 ± 1.08	0.025	0.126	0.138
Mean CHO_end_ (g·min^−1^)	1.46 ± 0.22	1.41 ± 0.35	−0.05 ± 0.28	−0.36 ± 2.26	1.42 ± 0.55	1.49 ± 0.43	0.06 ± 0.25	−0.03 ± 0.49	0.932	0.899	0.459
Mean HR (b·min^−1^)	133 ± 14	128 ± 11	−5 ± 9	−0.37 ± 1.33	123 ± 7	119 ± 7	−4 ± 5	−0.80 ± 0.51	0.078	0.036	0.838
HR at 120 min (b·min^−1^)	137 ± 14	131 ± 11	−6 ± 11	−0.43 ± 1.52	126 ± 8	120 ± 7	−6 ± 8	−1.01 ± 0.73	0.038	0.035	0.978
PFO (g·min^−1^)	0.57 ± 0.20	0.61 ± 0.30	0.05 ± 0.14^b^	0.56 ± 0.88	0.60 ± 0.20	0.50 ± 0.14	−0.09 ± 0.12^b^	−0.50 ± 0.44	0.762	0.567	0.046

Significance is inferred when *p* ≤ .05. Cohen's *d* effect sizes (ES) are presented ±95% confidence limits. Data derived from the incremental exercise tests feature nine subjects in the temperate training group and eight subjects from the heat training group. Performance time‐trial data compare seven subjects from the temperate training group and seven subjects from the heat training group.

Abbreviations: CHO_end_, endogenous carbohydrate oxidation; CHO_exo_, exogenous carbohydrate oxidation; CHO_tot_, total whole‐body carbohydrate oxidation; GE, gross cycling efficiency during the 120‐min constant‐load phase of the time‐trial; PFO, highest observed rate of fat oxidation during the incremental exercise test.

^a^ Indicates significantly different versus pre‐training.

^b^ Indicates significantly different between‐groups.

### 3.4 Muscle adaptations

**FIGURE 5:**
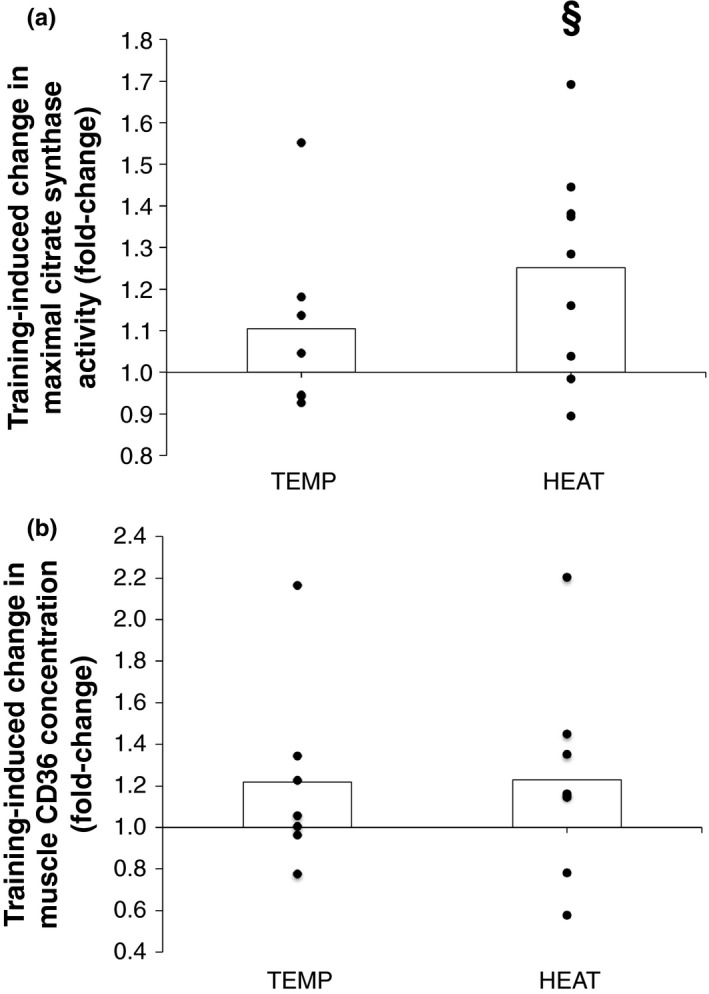
Training‐induced change in *vastus lateralis* (a) maximal citrate synthase (CS) activity and (b) cluster of differentiation (CD36) protein content in TEMP (*N* = 7) and HEAT (*N* = 9). Bars indicate mean pre‐ to post‐training fold‐change values and dots indicate individual responses. ‘§’ indicates significant within‐group change pre‐ versus post‐training (*p* ≤ .05)

A significant main effect of time was observed for maximal *vastus lateralis* CS activity (*p* = .007), but there was no group × time interaction (*p* = .149). Pairwise comparisons detected a significant increase in maximal *vastus lateralis* CS activity in HEAT (fold‐change, 1.25 ± 0.25, *p* = .03) but not in TEMP (fold‐change, 1.10 ± 0.22, *p* = .22). The magnitude of the training‐induced change in maximal *vastus lateralis* CS activity was not significantly different between‐groups (*p* = .25, Figure 5a), but was significantly correlated with the training‐induced change in 30‐min TT performance (*r* = .51, *p* = .04). No significant main effects of group (*p* = .59) or time (*p* = .09), or group × time interaction (*p* = .97), were observed for *vastus lateralis* CD36 concentration per unit of muscle protein (Figure 5b). Specific comparisons also did not reveal significant training‐induced changes in *vastus lateralis* CD36 concentration in TEMP (pre‐training, 15.7 ± 16.3; post‐training, 19.1 ± 20.3 pg·µg^−1^ protein, *p* = .14) or HEAT (pre‐training, 20.1 ± 11.5; post‐training, 23.3 ± 13.1 pg·µg^−1^ protein, *p* = .34).

## 4 DISCUSSION

The primary aim of the present investigation was to determine the effect of a three‐week endurance training intervention performed in 33°C on metabolism and performance in temperate conditions. The main findings were that (i) training performed in 33°C improved pre‐loaded 30‐min TT performance in temperate conditions to a significantly greater extent than matched training performed in 18°C, (ii) maximal *vastus lateralis* CS activity significantly increased following training performed in 33 but not 18°C, and (iii) a significant group × time interaction was observed for PFO during fasted incremental exercise. This study adds to the existing literature observing endurance performance in temperate conditions following periods of endurance training under heat stress, and provides the first systematic evaluation of the effects of moderate environmental heat stress during endurance training on metabolic adaptations. The results presented here warrant verification in larger studies, in elite and female populations, as well as in response to alternative training methodologies.

The training interventions in this study were completed successfully, with all participants completing all 15 prescribed sessions. As expected, relative power output achieved during moderate‐ and severe‐intensity training sessions was lower in HEAT compared to TEMP (Figure 2b,c), although by the end of the intervention power output during moderate‐intensity sessions was not significantly different between‐groups, indicative of heat acclimation (Figure 2b). Reduced mechanical training stress when under moderate environmental heat stress can be easily explained by the increased HR observed at a given power output (Nybo & Nielsen, 2001; Rowell et al., 1966), and reduced power output achieved at given physiological thresholds (James et al., 2017; Lorenzo et al., 2010). Therefore, in the present study, where training interventions in TEMP and HEAT were performed at matched relative cardiovascular load alongside ‘best‐effort’ severe‐intensity interval training sessions, reduced mechanical training stress was expected in HEAT. Importantly, RPE‐based training load and self‐reported well‐being were not significantly different between‐groups (Figure 2a). This indicates the training interventions were successfully matched, therefore allowing between‐group effects to be attributed to the addition of moderate environmental heat stress in HEAT. Interestingly, there appeared a tendency toward increased day‐to‐day variability in HRV in the second training week in HEAT versus TEMP (39 ± 19 vs. 23 ± 14%, respectively, *p* = .08, Figure 2d), which may be indicative of greater modulation of cardiac‐autonomic balance, and therefore a physiological stress response, in HEAT (Flatt & Howells, 2019; Javaloyes et al., 2019).

In line with our hypothesis, power output during a 30‐min TT preceded by two hours of constant‐load cycling and performed in temperate conditions significantly increased in TEMP and HEAT, but to a significantly greater magnitude in HEAT (30 ± 13 vs. 16 ± 5 W, *p* = .04, Figure 3, Table 2). These between‐group differences were observed only after the erroneous data‐point was removed (Figure 3a). A significant training‐induced increase in maximal *vastus lateralis* CS activity, a marker of mitochondrial protein content (Larsen et al., 2012), occurred in HEAT but not TEMP, which is a novel observation (Figure 5a). Our performance data align with recent research reporting significant improvements in 15‐min temperate TT performance in a group of elite cyclists performing 4–5 sessions per week for five weeks in 37.5–38.5°C (Δ ~19 W), but not when training at 15.5°C (Rønnestad et al., 2020). In that study, performance‐enhancing effects of training under heat stress were attributed to significant positive effects on hemoglobin mass not observed in the temperate group. Increased hemoglobin mass increased ${\dot{V}O}_{2}\text{max}$ (Rønnestad et al., 2020), which is unsurprising given O_2_ delivery appears limiting to ${\dot{V}O}_{2}\text{max}$ (Mortensen et al., 2005). In the present study, training‐induced changes in ${\dot{V}O}_{2}\text{max}$ were not observed (Table 2). However, hematological parameters were not measured and therefore cannot be dismissed.

The significant training‐induced increase in maximal *vastus lateralis* CS activity observed in HEAT but not TEMP is intriguing in the context of the lower mechanical work rates during training in HEAT (Figure 2b,c), and the association between training‐induced changes in *vastus lateralis* CS activity and 30‐min TT performance (*r* = .51, *p* = .04), although it should be acknowledged that the group × time interaction was not significant (*p* = .149). Endurance training performed under moderate environmental heat stress may augment expression of various factors implicated in the coordination mitochondrial biogenesis (Hawley et al., 2018). Accordingly, it could be hypothesized that exposure to moderate environmental heat stress during training in HEAT interacted with gene expression, protein activation, and/or protein chaperoning implicated in the adaptive mitochondrial response to exercise, though it should be acknowledged that these mechanisms are speculative. It is recommended that future, larger studies seek to verify our observations, and investigate the acute signaling mechanisms that may regulate this effect.

In terms of adaptations to whole‐body metabolic responses to fasted incremental and fed‐state, constant‐load exercise, the effect of environmental heat stress during training was minimal (Table 3, Figure 4). However, adaptation to PFO during the fasted incremental tests was significantly different between‐groups, with the training‐induced change significantly lower, and indeed negative, in HEAT (Table 3). Trained athletes tend to exhibit greater PFO than untrained individuals (Maunder et al., 2018), and thus the prevailing paradigm is that endurance training increases fat oxidation rates at given work rates. The possibility of blunted PFO adaptations to HEAT could plausibly relate to lower utilization of, and therefore adaptation to, fatty acid metabolism pathways during training (Febbraio et al., 1994; Hargreaves et al., 1996). Adaptations to whole‐muscle CD36 protein content, a sarcolemmal and mitochondrial membrane fatty acid transport protein, were not observed in either group, although it remains possible adaptations may have been observed at other levels of fatty acid metabolism. Therefore, the effects of endurance training performed under heat stress for acute metabolic responses to exercise in temperate conditions, and whether they are ergogenic or maladaptive, merit further investigation.

From an applied perspective, the results of this study provide tentative support for use of block periods of moderate environmental heat stress during training as a means of improving endurance performance in temperate conditions (Hawley et al., 2018). The observed 30‐min TT performance changes following HEAT are considered substantial (Figure 3a), particularly in the context of an already endurance‐trained population, who have higher reliability when performing TT assessments (Hopkins et al., 2001), and likely greater resistance to short‐term training‐induced changes in performance (Rønnestad et al., 2020). The perceptual and HRV data presented here also indicate that this training methodology can be effectively utilized without large perturbations to athlete well‐being or inducing maladaptation, which has occurred previously (Reeve et al., 2019). It is important to recognize the specific duration, training program, athletic population, and magnitude of environmental heat stress may have been critical to the adaptive responses observed here, and therefore longer training blocks or hotter conditions may not produce the same responses. It should also be acknowledged that the small sample size of this study prevents definitive conclusions being drawn from the data. Accordingly, it is suggested that future, larger studies should look to assess these responses using different training programs, elite and female athletes, and in different magnitudes of environmental heat stress.

## 5 CONCLUSIONS

In summary, a three‐week endurance training intervention performed in 33°C produced significantly greater positive effects on pre‐loaded 30‐min TT performance in a temperate environment than a matched training program performed in 18°C. This effect may have been at least partially attributable to beneficial effects of moderate environmental heat stress on *vastus lateralis* CS activity. This study therefore provides tentative support for the use of moderate environmental heat stress during a well‐controlled endurance training intervention for upregulating adaptations relevant to performance in temperate conditions, though further studies are warranted to verify and extend these observations. Moreover, programming training under heat stress via HR thresholds appeared an effective means of managing training stress. It is recommended that future studies assess the effect of different combinations of training and environmental heat stress on temperate performance adaptations including mitochondrial biogenesis.

## CONFLICT OF INTEREST

The authors declare no competing interests associated with this manuscript.

## AUTHOR CONTRIBUTIONS

Data were primarily collected and analyzed at the Auckland University of Technology laboratory at AUT Millennium; mass spectrometry was performed at the University of Waikato Stable Isotopes Unit. E.M. was responsible for original conception and design of the study, conducting the research at all stages, and original drafting of the manuscript; D.J.P. and A.E.K. were involved in the original conception and design of the study, interpreting the data, and revising the manuscript; G.A.W., M.J.B., W.L., W.L.C., and C.M.W. were involved in the design of the study, analysis and/or interpretation of the data, and revising the manuscript. All authors approved the final version of the manuscript and agree to be accountable for all aspects of the work in ensuring that questions related to the accuracy or integrity of any part of the work are appropriately investigated and resolved. All persons designated as authors qualify for authorship, and all those who qualify for authorship are listed.

## CONSENT TO PARTICIPATE

All participants provided written informed consent.

## CONSENT FOR PUBLICATION

All participants were informed of the intention to publish data generated in this study prior to providing informed consent.

## Data Availability

Data associated with this study are available from the corresponding author upon reasonable request.
